# Cross-Sectional Study on Oral Health-Related Quality of Life Using OHIP-14 in Migrants Children in Melilla (Spain)

**DOI:** 10.3390/children10071168

**Published:** 2023-07-05

**Authors:** David Ribas-Pérez, David Sevillano Garcés, Diego Rodriguez Menacho, Paloma Villalva Hernandez-Franch, Ignacio Barbero Navarro, Antonio Castaño Séiquer

**Affiliations:** 1 Department of Stomatology, University of Seville, 41004 Sevilla, Spain; drmenacho@us.es (D.R.M.); acastano@us.es (A.C.S.); 2 Independent Researcher, 41004 Seville, Spain; davidsevillanogarces@gmail.com; 3 Servicio Andaluz de Salud, 41004 Andalucia, Spain

**Keywords:** OHIP-14, melilla, migrants, oral health

## Abstract

Quality of life is a parameter that not only evaluates clinical parameters, but also refers to the perception of the individual in his or her sociocultural context. It also refers to psychosocial aspects that have a very important impact on people’s lives. Oral health-related quality of life (OHRQoL) must also be considered when assessing oral health in any population for which an oral health program is to be developed. On this premise, and taking into account the precarious situation of refugee children housed in the Temporary Center for Migrants (CETI) in Melilla (Spain), a study was conducted to assess the oral health and OHRQoL of the children housed in the aforementioned facility. For this purpose, the 120 children in care at the time of the study underwent a basic epidemiological examination according to the recommendations of the World Health Organization (WHO), and their OHRQoL was assessed using the specific OHIP-14 questionnaire. The results show a state of oral health with a high caries prevalence (95%), high DMFT, and dft indices for the studied population. The oral health-related quality of life perceived by these children shows that pain and psychological discomfort are the areas rated worst by them. Thus, it is concluded that it is important to implement specific oral health programs for this population, based on caries prevention and pain management, which must also take into account the psychological and sociocultural aspects that have accompanied their lives.

## 1. Introduction

WHO (1994) defines “quality of life” as the perception of the individual’s position in life within the cultural context and value system in which one lives and in relation to one’s goals, expectations, norms, and concerns [1]. It is a multidimensional and complex concept that includes personal aspects such as health, autonomy, independence, and life satisfaction, as well as environmental aspects such as support networks and social services [2].

Among the facets measured for the quality of life assessment, there are different domains (physical, psychological, degree of personal independence, social relationships, sociocultural environment, and spiritual level) [3,4,5].

In the field of oral health, there is also the term oral health-related quality of life (OHRQoL). OHRQoL is a relatively new concept that considers the individual’s self-perception of oral health as a component in addition to their clinical dental examination [6].

Oral health is closely related to an individual’s overall health and quality of life, as it affects oral functions and social interactions. For example, dental caries may be the cause of poor chewing, decreased appetite, sleep problems, and decreased academic and occupational performance or even social relationships [7,8].

On the conceptual basis of subjective measures with a broader perspective on oral health, the concept of OHRQoL has been developed to assess the extent to which oral health affects people’s behaviour and social functioning, complementing traditional clinical assessments of oral health [9,10].

Studies show that the impact of oral diseases and problems on OHRQoL is high worldwide. Some measures of OHRQoL have now been validated for specific populations in different countries. It is now important to consider their potential application in clinical practice [8,9].

There are several instruments available to measure oral quality of life. These include the Social Impacts of Dental Disease (SIDD) formulated by Cushing and colleagues in 1986, the Dental Impact Profile formulated by Strauss and Hunt in 1993, the Subjective Oral Health Status Indicators proposed by Locker and Miller in 1994, the Dental Impact on Daily Living (DIDL) proposed by Leao and Sheiham in 1996, the Oral Health-related Quality of Life (OHRQOL) proposed by Kressin and colleagues in 1996, and the Oral Health Impact Profile with its derivatives such as the children and adolescents COHIP version [10,11,12].

In the present study, the OHIP-14 questionnaire was used. OHIP-14 is a 14-item questionnaire that measures the limitations and discomfort that oral diseases cause in people’s lives. Ravaghi et al. demonstrated that it has the same reliability for the paediatric population as the COHIP, which may be more specific for children [13].

The OHIP-14 allows quantification of oral health outcomes in relation to patient quality of life by assessing seven dimensions: functional limitation, physical pain, psychological discomfort, physical disability, psychological disability, social disability, and obstacles [14,15].

In recent years, studies have been conducted using OHIP-14, which allows subjective quantification of aspects related to the impact of oral health on quality of life using scores at the end of each question [16]. The OHIP has been translated into many languages: Chinese, Korean, Japanese, French, German, Spanish, Portuguese, Malay, Arabic, etc. [17,18,19,20].

Refugees have different physical and mental health needs that are shaped by their experiences in their country of origin, their migration route, the host country’s entry and integration policies, and living and working conditions. These experiences may increase refugees’ vulnerability to chronic and infectious diseases [21,22,23].

The health of refugees and migrants is also closely related to the social determinants of health, such as employment, income, education, and housing. Refugees and migrants remain among the most vulnerable groups in society and often face xenophobia, discrimination, poor living, housing, and working conditions, and poor or limited access to general health services [24].

This is no different in the field of oral health. In their 2016 study, Reza et al. found that the children of refugees and migrants are more likely to suffer from dental disease. They are less likely to seek dental care due to language, cultural, and financial barriers [25].

We want to develop an oral health program in Melilla, a small Spanish enclave in North Africa that is a gateway to the European Union for many sub-Saharan migrants. In fact, Melilla is considered the second most used route for refugees trying to reach Europe [26].

The CETI (Centro de Estancia Temporal de Inmigrantes) is located in this city and was established to receive refugees who manage to cross the Melilla fence, as well as those who enter through authorised border crossings. It was inaugurated in 1999, when the phenomenon of immigration became more and more evident [2].

This centre was originally designed for 350 people and was later expanded to a maximum capacity of 480 people [27]. At the time this study was conducted, it housed about 1500 people from different countries, all with different customs, beliefs, and languages, and with the only common goal of reaching Europe as their final destination. A recent study has already highlighted the poor state of oral health of the children in this refugee centre [28].

With the aim of determining the oral health status and oral quality of life of the children housed at the CETI, an oral health study was conducted and related to OHRQoL to provide a situation analysis that would lead to the implementation of a specific oral health program.

## 2. Materials and Methods

### 2.1. Study Design

This is a descriptive cross-sectional study that examined the oral health of refugee and migrant children in the CETI. In designing the study, we followed the recommendations of WHO and used an oral health form based on the proposed model, which ensures standardisation of the results and comparison of the data obtained in this study with others [29].

In addition, oral quality of life was assessed using a simplified 14-question OHIP questionnaire, which was used in both French and Arabic, since the target population of the study was the refugee children at the CETI, who were predominantly of Arab or sub-Saharan origin. This questionnaire was validated through a pilot test, which did not reveal any ambiguities or difficulties in filling it out.

### 2.2. Individuals, Instruments, and Material Used in the Examinations

The study was conducted on 120 children between the ages of 4 and 16 years. At the time of the study, they corresponded to the totality of children housed in the institution, so there was no sample selection for the study.

The materials recommended by WHO were used for the study: essentially, a disposable flat mouth mirror, a wooden tongue depressor, paper towels, plastic bags, and protective materials such as gloves, masks, goggles, etc.

### 2.3. Personnel and Organisation

The oral examination was performed in a recumbent position, and a complete oral examination was performed by filling out the oral health form. Then, together with a bilingual translator (Arabic-, French-, and Spanish-speaking), the OHIP-14 questionnaire was completed for each child to facilitate the investigator’s communication with non-Spanish-speaking children. The assessments were conducted by a single investigator (D. S-G) who underwent pre-calibration with a kappa intra-observer concordance index of 0.92.

### 2.4. Variables Studied and Statistical Methodology

The condition of the oral cavity was studied using the indicators and criteria established by WHO, focusing on caries and dental malocclusion. The prevalence of caries and the indices dft and DMFT were examined, as well as the presence or absence of dental malocclusions and their expression as mild, moderate, or severe. Similarly, the questions of the OHIP questionnaire in its simplified version (OHIP-14) were evaluated [15,16].

The statistical methodology used was descriptive statistics with percentages, means, and the corresponding standard deviation. The Fischer test and chi-square analysis were used to cross variables with statistical significance when the *p*-value was less than 0.05.

## 3. Results

### 3.1. Oral Health

#### 3.1.1. Analysis of Age, Sex, and Country of Origin

The sample studied consisted of 120 children between the ages of 4 and 16 years, with an average age of almost 11 years (10.97 ± 3.43). The sample was almost balanced in terms of gender (53% boys, 47% girls), with the majority of the children studied being refugees of Syrian origin (Table 1).

#### 3.1.2. Caries Prevalence

Of the 120 children examined, 95% of the total had some form of caries, while only the remaining 5% were free of the disease.

#### 3.1.3. DMFT Index

The mean DMFT for the entire sample was 2.13 ± 1.9. The highest percentage (31.7%) corresponds to children with no caries experience in their permanent teeth (DMFT = 0), followed by 20% with a DMFT of 4, and 15% with a DMFT of 3 in their permanent teeth. The DMFT index at 12 years of age was 3 ± 2.6 (age to be considered by the WHO as a cohort to be considered in the comparative studies), which, according to WHO criteria, corresponds to moderate values.

#### 3.1.4. dft Index

The highest percentage, 21.7%, corresponds to children with no experience of caries in their primary teeth (dft = 0), followed by 20% with only one experience of caries in their primary teeth (dft = 1). The mean dft index corresponds to 2.43 ± 2.1 among the entire sample of children aged 4–6 years (children with complete primary dentition), which, according to WHO criteria, corresponds to the high values (Table 2).

#### 3.1.5. Malocclusion

The WHO recommendations were followed with regard to the classification of patients according to whether they had no malocclusion, mild, moderate, or severe malocclusion, with percentages of 41.7%, 55%, and 3.3% respectively.

### 3.2. Oral Health-Related Quality of Life (OHRQoL)

The reduced 14-question Oral Health Index Profile (OHIP-14) questionnaire measures responses on a Likert scale (0–4) with the following values:

Never (0), Hardly ever (1), Occasionally (2), Frequently (3), Very frequently (4).

To obtain a score, the points for each response are summed so that each patient can score a minimum of 0 points and a maximum of 56 points. Low scores indicate better self-perceived quality of life for the child, and high scores indicate worse self-perception of oral quality of life [15].

**Figure 1:**
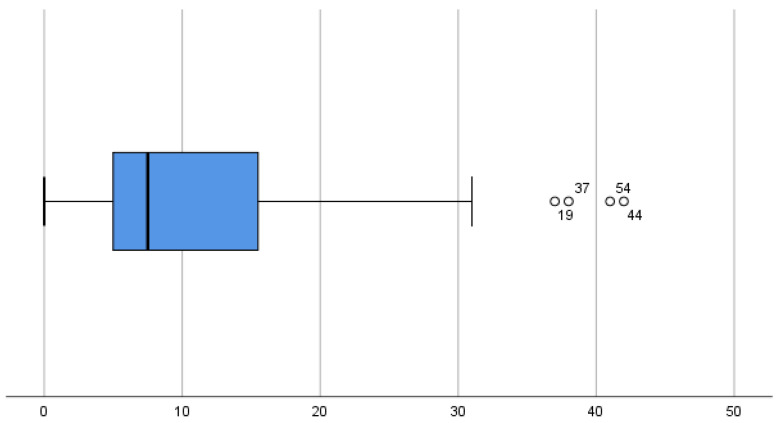
Final OHIP-14 cluster plot for the sample.

The overall mean OHIP-14 score for all patients was 11.25 with an SD of 10.99, resulting in a mean OHIP-14 score with an acceptable self-perception of oral health, as shown in Figure 1. From the same graph, it can be seen that the median is even lower than 10 (7.50), so more than 50% of the respondents have very good scores in self-perception of their oral health.

The results of the individual questions are shown in Table 3, where some problems with the pronunciation of words, pain, or even the proportion of interrupted meals in everyday life stand out. Of particular importance is the fact that almost 10% of respondents very often felt tense because of their mouth problems in relation to question 6.

This questionnaire provides the opportunity to examine problems such as functional limitations, physical pain, functional, or psychological discomfort, and others in groups (Table 3), and each of these problems has some weight in the calculation of the final score.

The greatest importance is given to physical pain with a score of 2.78, representing 24.7% of the total score, followed by psychological complaints, and the field that plays the least role is disability with a score of 0.70, representing 6.22% of the total OHIP-14 score.

### 3.3. Cross-Tabulation of Variables

**Figure 2:**
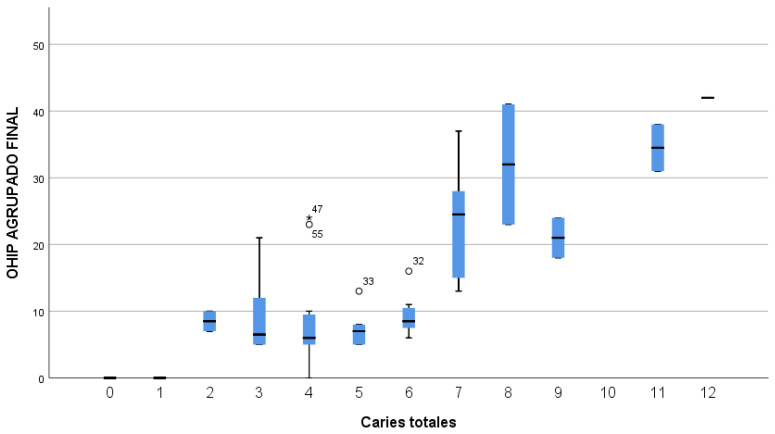
The relationship between the number of caries in children and the final OHIP-14 score.

When we relate the data from the OHIP-14 individualised questions to the prevalence of dental caries, we tested whether there was a relationship between the answers given and the presence of dental caries in the individual. This analysis showed that there was a statistically significant relationship between questions 3, 4, 7, and 8 and the presence of caries (*p* < 0.05), while there was no such relationship for the remaining questions (Table 4). It can also be seen that the higher the total number of caries, the worse the self-perception of oral health (Figure 2)

**Figure 3:**
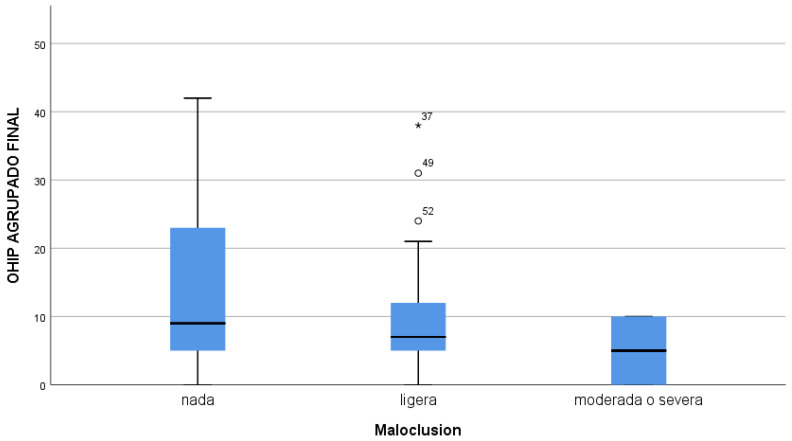
Relationship between degree of malocclusion and final OHIP score14.

Regarding malocclusion, there did not appear to be a relationship between higher levels of malocclusion and poorer self-perception of oral health in the sample, although there was a very small percentage of children with moderate or severe malocclusion in the sample. There was also no statistically significant association between any question of the OHIP-14 and the presence or absence of dental malocclusion (Table 4 and Figure 3).

In relation to the rest of the variables studied (country of origin, sex, or age), no statistically significant relationship was found with the OHIP-14 values.

## 4. Discussion

For this study, the final sample size was 120 children, and we focused on comparing oral health status and its relationship to perceptions of oral quality of life among migrant children housed at the CETI.

Our perception upon arrival at the centre is that these children have not had an easy life during their short life experience: they arrive in Melilla in deplorable conditions, with hardly any clothes, without any resources, and in some cases with poor nutrition. None of the children studied reported receiving any type of dental care during their long journey to the North African city, and medical care was strikingly poor [30].

The exploratory methodology used in this study followed the criteria of WHO on Basic Methods of Oral Surveys [29], and we decided to investigate dental caries and malocclusion. Other clinical indices were not included in the exploratory methodology for reasons of convenience, so we focused only on the pathology that we thought might have the greatest impact on oral health-related quality of life (caries and dental malocclusion).

Of the clinical caries variables, the DMFT index of the sample is 3 ± 2.6 teeth, which corresponds to a moderate caries index according to the criteria of WHO. These data contrast with the low caries level assigned by WHO in its recent study of 12-year-old children in Spain and Syria [31,32].

In Spain, the most recent study is from 2020 and shows a much lower DMFT of 0.58 ± 1.13 for 12-year-old children, which is significantly lower in the host country of these children [33].

In Syria, Hashish et al. [34] observed a 61% prevalence of caries and a dft of 3.3 ± 3.7 teeth in children aged 5–6 years, while, in the sample of refugee children from the CETI (majority from Syria), there was a dft of 4.8 ± 2.6 teeth in children aged 4–6 years with a prevalence of 95% of children with caries.

The relationship between the family environment and the acquisition of appropriate health habits and thus a lower prevalence of certain diseases, including caries, has been studied in detail [35]. In the situation of refugee children, families often break up and there is a lack of stability for establishing health and hygiene habits. This may have an impact on poor oral health, which is evident.

Beiruti et al. studied a group of 12-year-old Syrian children who had a caries prevalence of 74% and a DMFT score of 2.3, values that were lower but not as high as refugee children in the CETI [36].

Regarding the Spanish oral health targets for 2015/2020 for children, we can note that they are far from the results expected for Spanish children, as the DMFT index at age 12 years should be ≤1.0 teeth, as opposed to 3 ± 2.6 teeth for migrant children [31].

The same goals for the dft index in the primary dentition said that teeth with caries experience should have a mean of 2.4 at age 6 years. In the case of the CETI, this target is not met either, as the displaced children had a worrisome value of 4.8 ± 2.6 teeth, and the absence of fillings or previous dental experience is alarming.

Thus, it can be said that the dental care of these displaced children was conspicuous by its absence until their arrival at the CETI. There, it was possible to meet these basic health needs through the CETI in the Melilla Dental Clinic.

Under these conditions, it is necessary to implement a special oral health program for these children at the CETI, whose main objective is caries prevention and oral health education, which has long been proven by various studies [37,38].

Second, the reduced 14-question Oral Health Impact Profile (OHIP-14) questionnaire was used. The OHIP is an important tool for understanding oral health needs and developing strategies to control and reduce oral pathologies and promote oral health. It was selected because of its simplicity, the fact that validated versions exist in Spanish, French, and Arabic, and the fact that it is also valid for children, as shown by Ravaghi et al. [13].

A systematic review comparing the OHIP with other instruments measuring oral quality of life related to malocclusion also showed similar results in this regard, which led us not to select the OHIP-14 for our survey [39].

We decided to conduct the parent surveys in children aged 4–6 years, assuming that they were too young to answer the OHIP questionnaire conclusively. From the age of 7 years, the interviews were conducted directly with the children, provided that they exhibited mature behaviour, as has been the case in many studies that used OHIP as an instrument [40].

According to the results of the interviews, children generally had a very good self-perception of their oral health; however, we found that, in children with caries and with the increasing number of caries, the OHIP score was significantly higher, which seems logical. Similar results were found by Alves Ferreira in a study of 12-year-old school children in Brazil that used the same OHIP [41].

In general, oral health problems in refugee children do not have a greater impact on their oral quality of life than toothache, sensitivity, and irritability and discomfort typical of dental pain. We can therefore conclude that toothache is the parameter that has the greatest impact on the oral quality of life of these children, rather than caries as such.

A clear example of a process that has a lasting impact on people’s lives is dental caries, which can begin in early childhood in the form of early childhood caries and subsequently affect an individual’s entire life in many ways, not only through the pain it can cause. In fact, previous caries experience has been shown to be the most important predictor of poor oral quality of life, not only in the paediatric population [42,43]. A study conducted by Sinha et al. in 2020 in Indian children and adolescents has already indicated a clear association between poor oral health and impaired quality of life [44]. In this way, studies could continue beyond a cross-sectional study such as this one and be examined longitudinally over time.

The migrant child is aware of the fact that he has an oral health problem and can perceive it himself or herself, but has experienced psychological trauma in their country of origin and in the subsequent journey until their arrival at the CETI in Melilla and is able to cope with this oral health problem without it “affecting” his or her oral quality of life beyond the unavoidable, such as pain or sensitivity. In studies conducted in children with chronic diseases for whom life has not been very easy, we find similar data to our children in whom oral health remains in the background [45,46,47,48,49].

This is why in the vast majority of cases the poor state of oral health is not an impediment that stops the child carrying out day-to-day activities, as expressly asked in two questions of the OHIP test.

The other aspect that we thought could alter the OHRQoL of children was malocclusion. Although it is true that the measurement of malocclusion is very complex and that, in our study, we only used what WHO recommends as non-existent, mild, or moderate-severe malocclusion, which could be analysed in a much more complex way; in our case, we did not observe any relationship between the existence of malocclusion and a worsening of oral quality of life. Quite the opposite of these data was found in the study by Sun et al. in 2018, in which, after studying 300 patients, it was found that malocclusion had a very negative effect on quality of life, altering this alteration above all on a psychological level, probably assimilated as a need for orthodontic appliances, which in our group was not so important [50]. A positive point in regard to orthodontic treatment and its influence on the improvement in OHRQoL was already presented in the meta-analysis by Andiappan et al. in 2015, which showed that children who underwent orthodontic treatment saw a significant improvement in their oral quality of life [51], although it is true that, in our study, there was no comparative group that had received orthodontic treatment or even a more complex treatment that could affect the OHRQoL [52].

## 5. Conclusions

With the data obtained, we can conclude that there is a poor state of oral health in refugee children in the CETI in Melilla. Despite their high caries rates, their perception of OHRQoL is not affected beyond the sensitivity or pain that can be caused by dental caries.

There is a need to promote awareness of good oral hygiene among these children through parents and educators at the CETI and to promote preventive programmes for the child population, not forgetting immediate dental intervention for them.

## Figures and Tables

**Table 1:** Age, sex, and country of origin of the children in the sample.

Age (Mean and SD)		10.97 ± 3.43
**Sex**	***n***	**Percentage**
Boys	64	53.3%
Girls	56	46.7%
**Country of origin**		
Syria	82	68.3%
Morocco	21	17.5%
Algeria	8	6.67%
Mauritania	4	3.33%
Senegal	2	1.67%
Iraq	2	1.67%

**Table 2:** Data for caries (prevalence, dft, and DMFT) and malocclusions.

	N	Percentage	Mean	SD
Caries prevalence (%)	114	95%		
dft (<6 years)			4.79	2.60
DMFT			2.13	1.99
DMFT 12 years	18		3.04	2.58
No malocclusion	50	41.7%		
Light malocclusion	66	55.0%		
Moderate or severe malocclusion	4	3.3%		

**Table 3:** Percentage of each question of OHIP-14 questionnaire. Mean and SD.

QUESTION	0	1	2	3	4	Mean	SD
FUNCTIONAL LIMITATION						1.60 (1 + 2)	
1. Have you had problems pronouncing words because of problems with your teeth or mouth?	23.3	65.0	10.0	1.7	0	0.85	0.98
2. Have you felt that your sense of taste has worsened because of problems with your teeth or mouth?	69.0	20.0	10.0	10.0	0	0.70	1.01
PHYSICAL PAIN						2.78 (3 + 4)	
3. Have you had painful aching in your mouth?	21.7	41.7	20.0	8.3	8.3	1.40	1.16
4. Have you found it uncomfortable to eat any food because of problems with your teeth or mouth?	23.3	38.3	20.0	13.3	5.0	1.38	1.13
PSYCHOLOGICAL DISCOMFORT						1.94 (5 + 6)	
5. Have you been self-conscious because of problems with your teeth or mouth?	48.3	18.3	20.0	10.0	3.3	1.02	1.19
6. Have you felt tense because of problems with your teeth or mouth?	53.3	20.0	16.7	1.7	8.3	0.92	1.24
PHYSICAL DISABILITY						1.83 (7 + 8)	
7. Has your diet been unsatisfactory because of problems with your teeth or mouth?	46.7	30.0	16.7	5.0	1.7	0.90	0.63
8. Have you had to interrupt meals because of problems with your teeth or mouth?	25.0	56.7	15.0	1.7	1.7	0.98	0.79
SOCIAL DISABILITY						1.65 (9 + 10)	
9. Have you been a bit irritable with other people because of problems with your teeth or mouth?	63.3	18.3	8.3	10.0	0	0.65	1.00
10. Have you had difficulty doing your usual jobs because of problems with your teeth or mouth?	36.7	40.0	13.3	6.7	3.3	1.00	1.04
PSYCHOLOGICAL DISABILITY						0.75 (11 + 12)	
11. Have you found it difficult to relax because of problems with your teeth or mouth?	73.3	16.7	10.0	0	0	0.37	0.66
12. Have you been a bit embarrassed because of problems with your teeth or mouth?	78.3	10.0	8.3	1.7	1.7	0.38	0.85
HANDICAP						0.70 (13 + 14)	
13. Have you felt that life in general was less satisfying because of problems with your teeth or mouth?	80.0	8.3	10.0	1.7	0	0.33	0.73
14. Have you been totally unable to function because of problems with your teeth or mouth?	81.7	6.7	6.7	3.3	1.7	0.37	0.89
TOTAL						11.25	10.99

**Table 4:** *p* values for each question related to prevalence of caries and malocclusion, * *p* < 0.05.

QUESTION	*p*-Value Caries	*p*-Value Malocclusion
FUNCTIONAL LIMITATION		
Have you had problems pronouncing words because of problems with your teeth or mouth?	0.46	0.58
Have you felt that your sense of taste has worsened because of problems with your teeth or mouth?	0.55	0.26
PHYSICAL PAIN		
Have you had painful aching in your mouth?	0.022 *	0.57
Have you found it uncomfortable to eat any food because of problems with your teeth or mouth?	0.035 *	0.26
PSYCHOLOGICAL DISCOMFORT		
Have you been self-conscious because of problems with your teeth or mouth?	0.49	0.31
Have you felt tense because of problems with your teeth or mouth?	0.59	0.57
PHYSICAL DISABILITY		
Has your diet been unsatisfactory because of problems with your teeth or mouth?	0.016 *	0.63
Have you had to interrupt meals because of problems with your teeth or mouth?	0.050 *	0.81
SOCIAL DISABILITY		
Have you been a bit irritable with other people because of problems with your teeth or mouth?	0.60	0.36
Have you had difficulty doing your usual jobs because of problems with your teeth or mouth?	0.24	0.25
PSYCHOLOGICAL DISABILITY		
Have you found it difficult to relax because of problems with your teeth or mouth?	0.56	0.30
Have you been a bit embarrassed because of problems with your teeth or mouth?	0.93	0.80
HANDICAP		
Have you felt that life in general was less satisfying because of problems with your teeth or mouth?	0.85	0.73
Have you been totally unable to function because of problems with your teeth or mouth?	0.95	0.72

## Data Availability

All data will be sent by corresponding authors if it’s required.
